# “Everything's a fight”: A qualitative study of the cancer survivorship experiences of transgender and gender diverse Australians

**DOI:** 10.1002/cam4.5906

**Published:** 2023-04-18

**Authors:** Karolina Lisy, Lucille Kerr, Michael Jefford, Christopher Fisher

**Affiliations:** ^1^ Department of Health Services Research, Peter MacCallum Cancer Centre Melbourne Victoria Australia; ^2^ Australian Cancer Survivorship Centre, Peter MacCallum Cancer Centre Melbourne Victoria Australia; ^3^ Sir Peter MacCallum Department of Oncology The University of Melbourne Parkville Victoria Australia; ^4^ Department of Nursing Research, Cabrini Institute Melbourne Victoria Australia; ^5^ College of Health and Biomedicine Victoria University Melbourne Victoria Australia

**Keywords:** health Services for transgender persons gender identity, healthcare disparities, neoplasms, survivorship, transgender persons

## Abstract

**Background:**

There is widespread recognition of the need to achieve equitable outcomes for all cancer survivors. This requires understanding of the experiences and outcomes of vulnerable groups. People who identify as sexually or gender diverse are known to be at risk of inferior cancer and survivorship outcomes, however, the posttreatment survivorship experiences of transgender and gender diverse (TGD) people have not been well studied. This study explored the survivorship experiences of people who identify as TGD, focusing on the physical and psychological aspects of the posttreatment survivorship phase and their experiences of follow‐up cancer care.

**Methods:**

A qualitative study of 10 TGD cancer survivors. Interviews were transcribed verbatim and data were analyzed by thematic analysis.

**Results:**

Six themes were generated from the data. TGD people reported (1) anxiety when attending appointments and avoidance of necessary follow‐up care, (2) experiences of transphobia and discrimination within oncology care settings and (3) lack of consideration of TGD identity by providers. Themes further describe (4) physical aspects of being both TGD and a cancer survivor, (5) absence of inclusive and diverse supportive care resources, as well as (6) positive growth after cancer.

**Conclusion:**

Approaches to mitigate these issues are urgently called for. These include training in TGD health for health‐care providers, inclusion of TGD health in medical and nursing curricula, processes to collect and use gender identity and preferred pronoun data in clinical settings, and development of TGD‐inclusive information and peer‐support resources.

## 1 INTRODUCTION

Transgender and gender diversity (TGD) is an umbrella term that describes people whose gender differs from their biological sex assigned at birth. Internationally, it is estimated that up to 2% of the population are a gender other than cisgender male or female^1^; in the United States (US), it has been estimated that 0.6% of people aged 13 years and older are transgender.^2^ It is known that TGD people face challenges when accessing health care, including discrimination by health‐care providers (HCPs) which can include refusal of care, and may encounter inadequately trained HCPs who are not informed about the health needs of TGD patients.^3^, ^4^

TGD identity is associated with disparity across a range of health outcomes, including mental health, with TGD people more likely to experience depression, anxiety, past suicide attempt, and self‐harm compared to their cisgender counterparts,^3^, ^5^ likely owing to collected experiences of discrimination, social exclusion, and marginalization.^6^ TGD people may also be at elevated risk of some cancer types due to higher rates of some risk factors, including tobacco and alcohol use, and HIV and HPV infection, combined with lower rates of cancer screening.^7^, ^8^ Indeed, there may be a higher incidence of infection‐related cancers in transgender versus cisgender people,^9^ however, knowledge of cancer incidence and mortality outcomes in TGD people is limited by the lack of population‐based studies and gender identity data collection by cancer registries.^8^

Cancer survivorship care aims to monitor for cancer recurrence and also to address any physical or psychological needs that may result from cancer or treatment, with widespread recognition of the need to achieve equitable outcomes for all cancer survivors.^10^ Integral to these efforts is understanding the experiences of “at risk” or “vulnerable” groups. Collectively, sexually or gender diverse people  (including but not limited to lesbian, gay, bisexual, transgender, and queer [LGBTQ+]) are known to be at risk of poorer cancer and survivorship outcomes and experiences of care compared with heterosexual, cisgender people.^11^, ^12^, ^13^ Within the LGBTQ+ population, very little is known specifically about the posttreatment experiences or outcomes of people who are TGD.

This qualitative study explored the survivorship experiences of TGD people who had been diagnosed with cancer and completed primary cancer treatment, specifically focusing on the physical or psychological aspects of the posttreatment survivorship phase and their experiences of follow‐up cancer care.

## 2 METHODS

### 2.1 Methodology

This qualitative study was part of a larger national Australian study, the Trans Health and Cancer Care Study,^4^ and received ethical approval from the La Trobe University Human Research Ethics Committee.

### 2.2 Participants

Participants for the Trans Health and Cancer Care Study were recruited between September 2018 and January 2019, primarily via Facebook advertising, as well as via posters displayed in TGD‐specialist GP clinics, an LGBTQ+ bookstore, and via a TGD radio program. Participants were invited to provide their email address if they had been diagnosed with cancer and wished to participate in an interview about their experiences. Eligible participants for this study were TGD adults (≥18 years), who resided in Australia, had a prior diagnosis of cancer, and had completed treatment.

### 2.3 Procedures

Interviews were conducted by one researcher (LK) either in person, or via telephone or video call (Skype). Both written and verbal informed consent to participate in this study was obtained prior to each interview. At the time of the interviews, LK was a PhD candidate and specialist cancer nurse with experience in conducting interviews with TGD people. Interviews were semi‐structured, utilizing broad open‐ended questions designed to allow participants to direct the conversation. The interviewer asked additional questions seeking further detail and clarity based on participant responses. Overall, 12 interviews were conducted, ranging from 44 to 143 min in length, with an average length of 76 min. Two participant interviews were excluded from this analysis as participants did not meet the criteria for a cancer survivor. Specifically, one participant had a BRCA mutation and prophylactic mastectomy, and one participant had a hereditary, benign cancer syndrome. These participant interviews were included in the broader study published elsewhere.^14^

### 2.4 Data analysis

Interviews were recorded and transcribed verbatim, and data were analyzed using thematic analysis. Transcripts were read in full and preliminary notes made prior to importing transcripts into NVivo for analysis. Transcripts were then reread and descriptive codes assigned to the text by one author (KL). Interviews were broad and included experiences of cancer diagnosis and treatment, however, only data describing experiences posttreatment were included in this analysis. Codes were continually refined throughout the data analysis process to generate the final coding framework that represented the breadth and depth of participant survivorship experiences. Codes were then analyzed to identify patterns in the data and develop themes, which were shared with the author team for refinement and agreement. Results of the thematic analysis are presented alongside direct participant quotes to provide evidence for and illustrate each theme. Participants are identified by code in the narrative (P1, P2 etc.), with gender identity provided for context (Table 1).

**TABLE 1:** Participant characteristics.

Participant	TGD identity	Age (years)	Cancer type
P1	Trans woman	38	Testicular cancer
P2	Trans man	60	Lung cancer and lymphoma
P3	Trans woman	38	Melanoma
P4	Trans woman	47	Prostate cancer
P5	Nonbinary	47	Breast cancer
P6	Nonbinary	18	Hodgkin's lymphoma
P7	Trans man	38	Basal cell carcinoma
P8	Trans woman	50	Bowel cancer
P9	Genderqueer	53	Basal cell carcinoma, melanoma and leukemia
P10	Trans man	26	Liver cancer (as infant)

### 2.5 Positionality statement

The lead author (KL) is a Senior Research Fellow with experience in both qualitative methods and research in the areas of cancer survivorship and sexual and gender diversity. KL is a cisgender woman and an LGBTQ+ ally. She conducted this work in the pursuit of equity in care and outcomes for TGD people affected by cancer, however, acknowledges that she approaches this study (necessarily) from an outsider's perspective, with any knowledge brought forward from professional rather than personal experiences.

## 3 RESULTS

Transcripts from 10 interviews were analyzed, with participants between 18 and 60 years old (Table 1). Participants had a variety of prior cancer diagnoses, and a diversity of gender identities. Thematic analysis yielded six overlapping themes, described below, alongside supporting quotes (Table 2).

**TABLE 2:** Themes and participant quotes.

Themes	Participant quote
Anxiety and avoidance	“*I tend to avoid doctors unless I absolutely need to go*.” (P7, trans man) “*I would just continue to put it off, and especially for like breast screening as well, I don't even foresee myself going at all, even though I know the importance of screening for cancer, I know it very intimately but I can't bring myself to go to those appointments*.” (P10, trans man) “*The worst part of the melanoma is taking your clothes off all the time, because in the public health system, whichever doctor I'm going to get changes every time… and I always have just a little bit of anxiety, because I never know who is going to be on the other end, you know, I've got a lot of tattoos and I look the way I look… every time I have to take my clothes off it's like ‘I don't want to take my clothes off’, followed by ‘I hope I get someone nice’*.” (P9, genderqueer)
Transphobia and trans‐competency in health care settings	“*I'm no longer re‐traumatised by being in a hospital, but I really don't like it much, and I also, I've also dealt with a lot of transphobia from doctors, so that's always a concern as well.*” (P7, trans man) “*It's been a real battle to get any help that's appropriate, and in‐home nurses that come, they just send you a new one, every single time it's a different person and if you can appreciate the anxiety of trying to get someone that you'll think you'll be safe with, um, and some were accepting and some weren't, some were really rough, yeah, when they sort of found out, and just walked off*.” (P2, trans man) “*On one of my blood tests they used a male title, so they called me ‘Mr [name]’, and I was like ‘fucking hell that's really shitty’, but you know, so that's pretty standard, the amount of mail that I get delivered to me that is like ‘Ms my dead name’ or Mr [name]*.” (P3, trans woman)
Consideration of TGD bodies by HCPs	“*…they [doctors] just would not listen to me in any way, like the whole, any mention of the word trans was like a film would go over their eyes and they couldn't hear it, it was just no listening to anything to do with it, to them it was just completely irrelevant, and I understand they've got a priority and they've got a specialist field, but you know, really, like my psychology was, you know, my mental psychology was in a complete mess*.” (P4, trans woman) “*Never in my life had anyone asked me, ‘do you actually want to repair your penis?’… that was the most ridiculous question that he could've asked, you know… just by the way he addressed me I could see he had absolutely no inkling of anything to do with a trans woman, like, no inkling of the sort of issues that you might face*.” (P4, trans woman) “*I said to him, ‘I said to you specifically, I made it clear to you that I was a post‐operative trans woman, things down below were different to a genetic female,’ you know, things had been fashioned there – in other words I wasn't self‐lubricating or anything like that, and I said, ‘you knew this, I made you aware of this and your only caution to me was that I might feel dryness’*.” (P8, trans woman)
Physical aspects of TGD body in survivorship	“*Obviously I'd like girl parts, that would be fantastic, oh that's another thing, yeah, I can't have a sex‐change operation because of the radical prostatectomy, I don't know if that's relevant to your study, because of scar tissue*.” (P4, trans woman) “*I think if anything, I think having breast cancer and the whole sequence of events to having no breasts, I actually think it's made me a lot stronger about what I identify as…it made me speed up the process, this is who you are, do you want to get rid of your breasts? Here we go*.” (P5, non‐binary) “*I was at home and I was recovering and I felt free of the breasts, and you know, I felt… I really was what I always wanted to be, flat‐chested, and I was really happy with the surgery.*” (P5, non‐binary) “*I think just being trans diminishes the significance of that sort of imperfection, because there's already so much imperfection that you're kind of just like ‘meh'."*(P3, trans woman) “*Pre‐puberty, I didn't feel comfortable about my scar, and then on the flip‐side of that when I started going through puberty I didn't want to take my top off or, you know, be in that kind of situation where anything was on show because I was developing breasts, I didn't care about my scar anymore.*” (P10, trans man)
Lack of TGD resources and support	“*…as a trans person I was just kind of, like, forgotten about, the language wasn't there, there wasn't information there.*” (P10, trans man) “*There's just nothing to meet up about, they're on a different planet than me, we've got different concerns. I'm not worried about what my husband thinks.*” (P2, trans man) “*…I went to a group, and it was good but I just felt like it was really straight and I didn't feel like I was part of it because nobody was queer, not that I needed it to be, but it was very straight, it was very heterosexual and I just didn't feel like a kind of connection, like I knew these women were talking about similar things but… I felt like I was such more a minority of individuals who never wanted their breasts back, yeah, I was very tomboyish looking so I just ended up, yeah, I found it really challenging, I didn't actually go to any more groups because I…just kind of didn't feel part of it, I did not feel the connection.*” (P5, non‐binary) “*I went there, as I say, a de‐transitioned woman, so I went there as a man with breasts, and a female partner, and men with breasts are not uncommon in prostate cancer support groups, if you're going to get a man with breasts, that's the place to look, because that's a side effect of the treatment of prostate cancer, the hormone therapy. So there's quite a few guys in there that are on hormone therapy and growing breasts, obviously they probably feel differently than me about it, but yeah, like, as I started my transition again, I could see that they were a bit weird, you know, they were a bit weird with me at the start.*” (P4, trans woman)
Positive growth after cancer	“*In a way it's made me more confident because beforehand I absolutely hated my body, I was self‐harming, I was suicidal, all that crap, I was just really not accepting of myself*.” (P6, non‐binary) “*I just stopped caring about anything, now I don't, yeah, I don't care about societal constrictions, I don't care what people think, I just think, like, stuff it, life's too short*.” (P4, trans woman) “*I guess I'm more confident in my ability to, you know, deal with that kind of stuff, surgery, advocate for myself, that kind of thing*.” (P7, trans man)

### 3.1 Anxiety and avoidance

A strong feature of the survivorship experience for TGD people was feeling stress or anxiety around attending follow‐up appointments, including necessary cancer screening. These feelings are specific to their TGD identity rather than or in addition to stress that may be felt due to their cancer history or fear of cancer progression. One participant described being trans in health‐care settings as “*kind of a constant low‐grade stress*” (P7, trans man), and another participant described the stress of attending follow‐up appointments as being in a state of hypervigilance “*because I'm always waiting for someone to pick me, someone to say ‘you're not a woman’, and that in itself is very tiring*” (P8, trans woman). Stress regarding cancer follow‐up for some participants was centered on needing to remove clothing during appointments (see Table 2).

Anxiety around attending medical appointments also leads to avoidance, with some TGD people saying they do not attend follow‐up at all, despite knowing the importance of cancer follow‐up care, or delay seeking care even when experiencing concerning symptoms: “*I was thinking, ‘just go, you've had that melanoma,’ so what I do is I procrastinate my health as a direct result of the [previous bad] experiences*” (P9, genderqueer). This is highly relevant for gendered screening services, where TGD people may feel particularly uncomfortable or conspicuous in gendered spaces: “*certainly after coming out as trans I have not wanted to go into a specified place for screening for that exact reason of being the only man in a female's ward*” (P10, trans man).

### 3.2 Transphobia and trans‐competency in health‐care settings

Anxiety surrounding follow‐up care may be due to expectations of discrimination or poor treatment, and based on prior transphobic experiences within health‐care settings. Transphobia was felt by TGD survivors in their interactions with some HCPs, including reception staff: “[Reception staff were] *short and sweet, not as friendly as the person who may have been in the line before me or the person who may follow me*” (P9, genderqueer). One participant felt that their surgical outcome was impacted by their gender identity and presentation, and that the surgeon had not taken the requisite care with removal of a facial BCC that they would have had the participant presented as a woman: “*Compared to the next guy who did my mouth, like, he would not have matched a woman's lips up like that, you know, like distinct, not that I don't love the scar but I just go, ‘that's what homophobia is,’ a ‘less‐than’ attitude that means the outcome is really different*” (P9, genderqueer).

Transphobic interactions also included verbal misgendering by HCPs, which made one participant feel like “*everything's a fight*” (P4, trans woman). Anxiety in health‐care settings may also result in people not wanting to disclose their gender or not correcting providers when they are misgendered. One participant described not requesting their preferred pronouns be used because “*I try to get in and get out with the least homophobic experience as I can*” (P9, genderqueer). Regarding asking for preferred pronouns to be used, another participant said they did not ask because “*I was really too scared to, I felt like I didn't have a choice, it was just go with what you're given*” (P6, nonbinary), even though being misgendered made them feel like “*you're talking to someone else*” (P6, nonbinary). Lack of inclusivity was also embedded in paperwork and administrative processes, where TGD identities were not accommodated. This included not having the required fields or options on patient records to input a patients' gender, pronouns, and preferred or chosen name, and not having processes in place to ensure that correct names and pronouns are used by HCPs, both verbally when attending services or in any subsequent documentation that is provided to the patient (see Table 2). Not recording and using preferred names was interpreted as an intentional action and rejection of TGD identity: “*…they want to use their position of power to let you know that they don't accept your gender identity, and that's what it is, whenever they say anything about refusing to accept your name. Because people will say, if you tell them, ‘oh this is my name, that's my legal name,’ they'll say, ‘okay, well I'll have to put that on the form, we'll put that as preferred name,’ done, simple, but, you know, there's people who just have to make a point of saying, ‘I don't accept your gender identity,’ and that's the thing, ‘oh sorry we have to use the name on your documents.’ No, they don't. That's just them saying ‘sorry, I don't accept your gender identity, I'm going to use this small bit of administrative power to stick it in your face,’ that's how it makes me feel*.” (P4 trans woman).

Most of the participants described transphobic encounters and interactions when attending cancer follow‐up care, however, one participant articulated the need for care that goes beyond being trans‐friendly to care that is competent in addressing the needs of TGD people. This participant described having to take on the role of educating HCPs: “*Because I'm now read as male, going and seeing a doctor for something like a Pap smear, that's always a bit weird and they never know like how the testosterone interacts, like they're not trans‐competent, they might be trans‐friendly but that doesn't mean that they're trans‐competent, so I have to do a lot of educating as well*” (P7, trans man).

### 3.3 Consideration of TGD bodies by HCPs

Maintaining TGD identity throughout and after cancer is critical to individuals' well‐being. Illustrating the essential nature of gender identity to TGD survivors, one participant described surviving childhood cancer as: “*I definitely felt guilty because they'd saved my life and now I'm like, ‘I don't really like this life, this isn't how I want it’*” (P10, trans man).

Despite the critical nature of maintaining TGD identity, participants described lack of consideration of their gender during and after cancer treatment. A common thread through participants' stories was unsatisfactory interactions with HCPs, particularly around overlooking their TGD status as an important part of their cancer experience, not considering the potential impact of cancer or treatment on their bodies, and making assumptions due to their TGD identity (see Table 2).

One participant who had undergone radiation therapy for bowel cancer described their distress at the physical side effects of treatment (“*I can't have sex. I spent years with gender dysphoria, not being happy with what was there, and then I get to the stage where I can finally fix that and then two years later I can't use the thing, yes I'm a little bit distressed about that*.” (P8, trans woman)) and the lack of discussion from HCPs regarding the impact of treatment on their body (“*at no point had anyone ever said anything other than, ‘you might suffer some dryness,’ so I was a little disappointed with that, not having been given the full story, not having that time to prepare for that mentally*” (P8, trans woman)).

For some TGD people, gender‐affirming hormone therapy is a key part of maintaining gender identity, and two participants described their experiences with hormone therapy during and after cancer treatment. One participant who ceased gender‐affirming hormone therapy during treatment for prostate cancer described the unwillingness of doctors to provide estrogen therapy as part of their treatment and their resulting “de‐transition” as highly distressing: “*I still remember looking at my face every day in the mirror, watching my, like, adipose fat distribution slowly dissipate, that was the worst experience of my life. I would never de‐transition again… that was the most painful part of it*” (P4, trans woman). Another participant was able to continue hormone therapy throughout treatment for testicular cancer, but did not receive follow‐up care with an endocrinologist: “*No, they didn't stop my hormones, normally basically what they would've done they told me…they would just put me onto an endocrinologist, and then they would've put me on testosterone but because I was already on oestrogen they didn't even bother with any of that. Although, I probably should go and see an endocrinologist, that should've been done years ago*” (P1, trans woman).

### 3.4 Physical aspects of TGD body in survivorship

There was substantial discourse regarding the physical aspects of living with and after cancer in a TGD body. Participants described a range of unique situations regarding how being TGD intersected with their cancer treatment and recovery, both positive and negative.

One participant described the challenge of being both trans and a cancer survivor, specifically regarding sexual dysfunction arising from cancer treatment: “*I wanted to transition and have a boyfriend, I've craved for that time and I am reluctant to do that now because of the cancer, because of the vaginal stenosis…. as a trans person, it's hard enough as a single woman at 50 to find a partner…then, if you find someone that they're a bit interested in you, then to have to have the trans conversation, which I think at some point you've got to have that conversation… now if the guy hasn't left you then, then you've got to have this conversation about not being able to function maybe properly sexually*” (P8 trans woman).

Some participants highlighted that cancer treatment interfered with gender affirming surgeries. In one case, a participant felt fortunate to have had cancer after transitioning: “*I was also lucky, in hindsight, that I had my reassignment surgery before cancer, if I had it, well if I'd had cancer first I wouldn't have been able to have gender reassignment surgery… had I not been able to have that, for whatever reason, life would not have been worth living at all*” (P8, trans woman).

For other participants with gender‐specific cancers, surgery that removed unwanted body parts was seen as beneficial and as a way of the body “*getting rid of a piece that's not supposed to be there*” (P1, trans woman). A participant with a history of testicular cancer stated: “*…my testicles had caused so many problems for me throughout my life, I should never have had them… It's definitely been really beneficial actually, having them removed, like I just feel normal, I suppose, for the first time in my life*” (P1, trans woman).

Further to this, many participants noted that due to their history with gender dysphoria, they were not bothered by surgical scars from cancer treatment, or liked their scars, as the experience of gender dysphoria was more distressing: “*I've got this, you know, big scar down here from the bowel surgery and that doesn't bother me, not a bit, and I think to myself, ‘why doesn't that bother you? That should bother you,’ I think ‘no’, my body now is the way it should be as far as my gender dysphoria was concerned, having a scar there – couldn't care less*” (P8, trans woman).

### 3.5 Lack of TGD resources and support

Participants described an absence of support that was relevant to them and their needs in the posttreatment phase, as well as a lack of information and resources where TGD people were represented: “*…they had racks and racks of every type of cancer brochure you could possibly imagine… there wasn't one single word or picture anywhere through every document that even addressed gay, let alone trans, there was nothing about it*” (P2 trans man).

Peer support services such as support groups and one‐on‐one support were often discussed, with participants highlighting the importance of peer support and the need to share experiences with people that had been through cancer. Peer support may be particularly important for trans people, who may have difficult or severed relationships with their families (“*I don't have all the back‐stops that people [usually have], you know, just normal family and the family thing*” (P2, trans man)), or be otherwise lacking in social support (“*Well I've been isolated, I'm a trans woman, I've been isolated and rejected all my life* (P4, trans woman) and “*It's definitely made it harder, like, not having, not having like friends that I could ring and talk about it*” (P7, trans man)).

The need for a “queer” support group for cancer survivors was voiced: “*I just felt like there was a need out there to be with like‐minded queers who have experienced it, you know, our experiences, I feel, are so different than the average person, you know, who is probably married and got children, I just feel that we, I'm a non‐binary within a minority that, yeah, I'm just, yeah, I felt that quite challenging and at times that I'm quite alone. I hope one day there's more groups or there's something out there*” (P5, nonbinary). Participants noted, however, that “*support networks for trans people with cancer are non‐existent*” (P1, trans woman) and when they did attend available support groups, participants described feeling out of place, or unable to connect with others (see Table 2).

### 3.6 Positive growth after cancer

The majority of participants described aspects of positive growth following their cancer experience, either in terms of their relationship with themselves and their bodies, relationships with others, or being more confident in themselves and assertive in their day‐to‐day lives.

In terms of individuals' relationships with their bodies, participants described treating their bodies with more respect and acceptance after cancer: “*What it's made me do is be really aware to, like, I don't drink, I don't take drugs, I've recovered from addiction issues a long time, 15 years, and I really try to treat myself well*” (P9, genderqueer). Connected to being more accepting of their bodies, many participants also described feeling more empowered to be themselves, and more confident in advocating for themselves (Table 2).

Some participants described fractured relationships with parents or their families due to their gender identity, and becoming closer due to cancer: “*[my parents] went from hating me to helping me, in the space of a weekend*” (P1, trans woman); and “*Certainly this whole quote‐unquote trans thing has always been um kind of rocky for mum because she doesn't, she didn't understand for a long time… but in terms of the cancer experience, yeah we're pretty close because of it, I think, we've got a lot of mutual friends because of it, so yeah it's been a positive thing for mum and I*” (P10, trans man).

## 4 DISCUSSION

An essential part of striving for equitable outcomes for all cancer survivors is understanding the experiences of marginalized or vulnerable groups. To this end, this article has described the lived experiences of TGD survivors of cancer, detailing themes around anxiety in health‐care settings, avoidance of follow‐up care, lack of competent care and experiences of discrimination and transphobia, the intersection between cancer treatment and TGD bodies and the need for HCPs to consider this, as well as positive growth after cancer.

This study's theme of anxiety and avoidance included TGD people who described not attending follow‐up appointments despite being fully aware that surveillance for recurrent or new cancers was a critical component of their survivorship care. Given that there may be disparities in a number of key cancer outcomes such as stage at diagnosis, receipt of treatment, and survival, for a number of cancer types for transgender people compared with cisgender people, avoidance of cancer follow‐up is a critical issue for this survivor group.^15^ Avoidance of cancer screening, and more broadly, avoidance of health services, has been described previously,^4^ and remains a prominent barrier to reducing inequality in cancer and other outcomes between TGD and cisgender people.

To begin to mitigate this issue, of immediate importance is the implementation of strategies which aim to improve the experience of TGD people when accessing cancer services (and more broadly health services in general). There was a strong emphasis on experiences of transphobia, discrimination, and lack of gender‐affirming care in this study, and other research similarly indicates that LGBTQ+ people with cancer feel unsupported within the health system.^16^ Misgendering patients in health‐care settings was commonly raised, and may cause patients to feel that their provider is either not interested in them or their health, is uncomfortable with their TGD identity, or is unwilling to provide care.^17^ Including a more complete range of gender identities within administrative processes, as well as enquiring about and respecting individuals' preferred pronouns, are relatively simple yet crucial steps which enhance TGD visibility in health settings and may provide a sense of safety for TGD patients. Collection of sexual orientation and gender identity (SOGI) data, in either clinical and research settings, has been found to be acceptable by TGD people, however, has not been widely adopted to‐date.^18^, ^19^ Moving forward, it is imperative that ongoing collection of SOGI data is implemented in oncology settings to not only facilitate appropriate clinical care, but also to enable monitoring of TGD cancer outcomes, experiences, and areas of disparity.

Importantly, alongside experiences of discrimination, participants described HCPs as lacking in cultural competency, with limited or absent discussion of their TGD status and how they as individuals may be impacted by their cancer or treatment. In agreement with these reports, a retrospective case series of 37 transgender patients with cancer revealed that only four had their preferred pronouns documented and only five included any documented discussion of their gender identity and how this may interact with their cancer treatment.^20^ Furthermore, transgender people often report having to “educate” HCPs about TGD health needs.^21^ An earlier systematic review of lesbian, gay, and bisexual people with cancer found that in the absence of homophobic interactions with HCPs, patients felt grateful for being treated equitably.^11^ It is telling that only one participant in this study articulated a need for trans‐competent care in addition to trans‐friendly care, which based on the experiences of our current cohort, is certainly lacking.

Australian data shows that a third of transgender people surveyed support funding for training doctors in TGD health.^3^ Research indicates that while HCPs may have lower levels of confidence when it comes to treating TGD people, they are keen to improve, however, barriers such as a lack of education opportunities exist.^22^, ^23^ These data collectively point to the urgent need for training around TGD health for current HCPs, and certainly for greater inclusion of TGD health in medical and nursing curricula moving forward.

Support for TGD cancer survivors and their carers may also be improved through greater availability of LGBTQ+‐inclusive information materials and LGBTQ+‐specific support groups. Our study findings are corroborated by others describing that LGBTQ+ people often feel out of place in mainstream cancer support groups,^11^, ^16^ leaving TGD survivors potentially missing out on the benefits of peer support. Having up‐to‐date knowledge of existing LGBTQ+ supportive resources that HCPs may suggest to TGD survivors may provide these patients with more appropriate options. A recent survey of transgender Australians indicated that 50% reported their preferred method of receiving health information was online,^3^ representing an avenue for information provision and creation of further peer support programs.

In response to this study's findings, suggestions for implementation and research are presented (Table 3). These include training and education in cultural diversity for all HCPs, including patient‐facing workers such as reception staff, inclusion or expansion of TGD health in medical and nursing curricula, implementing processes to collect and consistently use preferred names and pronouns, and developing and disseminating TGD‐specific cancer and survivorship information and resources. Patient‐centredness is key to improving care of TGD cancer survivors, and training and education may better equip HCPs to sensitively and competently provide care that addresses the holistic needs of TGD people. Asking about and using correct names and pronouns, and displaying information materials such as posters or pamphlets that reflect TGD identity or use recognizable symbols, may signal to a TGD person entering a health‐care setting that they are in a safe or TGD‐friendly space. Finally, more research is needed to understand the impact of cancer and cancer treatments on TGD bodies, particularly around the interaction with and impact of various treatment modalities on gender‐affirming surgery and hormone therapy. It is important that future research and the development, implementation evaluation of any interventions is done in genuine, meaningful consultation with the TGD community to ensure safety, inclusivity, and responsiveness to the needs of TGD people.

**TABLE 3:** Suggestions arising from this study for implementation and research.

Suggestions for implementation and further research: Develop, implement and evaluate TGD‐specific diversity training for current health‐care staff (including non‐clinical staff, i.e. reception staff) Include/expand training in TGD health in pre‐vocational medical and nursing curricula Develop, implement and evaluate processes for sensitively collecting and consistently using gender identity, preferred pronouns and preferred names in clinical settings Develop, disseminate and evaluate TGD‐specific information resources Provide broader peer support options for TGD people, which may include linking patients with existing LGBTQ+ peer support networks, or suitable online peer support programs Conduct research to understand the unique impacts of cancer and cancer treatment on TGD people, particularly the impacts of various cancer treatments on gender‐affirming care

### 4.1 Strengths and limitations

Very few studies have focused on the cancer experiences of TGD people, and even fewer have examined the posttreatment survivorship phase. To the best of our knowledge, this is the first qualitative study to focus on TGD cancer survivorship, without combining sexually and gender diverse people together. This study therefore addresses a critical knowledge gap, with data leading to suggestions for development of initiatives that may be implemented to improve care, support and experiences for this survivor group. We acknowledge a limitation of this study as a result of the recruitment approach, with potential participants responding to advertisements and opting to be contacted for interviews. As a result, those who responded to study recruitment information may include those who are more actively engaged in TGD spaces and communities, have social media access, and access TGD health services. Previous research has identified lack of trust in research and fear of being “outed” as barriers to TGD people participating in research studies^24^; therefore, it is possible that this study did not include voices of people who do not wish to be identified as TGD or those who may be uncomfortable with participating in research. This is an important limitation to acknowledge as these people may experience significant barriers and challenges when accessing cancer and survivorship care that may not be represented here.

## 5 CONCLUSION

Qualitative inquiry into the cancer survivorship experiences of TGD people has revealed experiences of discrimination within oncology care settings, anxiety when attending services and avoidance of necessary follow‐up care. Significant gaps in provider knowledge of TGD health, including the potential impacts of cancer and treatment on TGD bodies, and lack of appropriate supportive care information and other resources also featured. Approaches to mitigate these issues are urgently called for.

## AUTHOR CONTRIBUTIONS

**Karolina Lisy:** Conceptualization (supporting); formal analysis (lead); funding acquisition (equal); investigation (equal); project administration (equal); writing – original draft (lead); writing – review and editing (lead). **Lucille Kerr:** Conceptualization (equal); data curation (equal); investigation (equal); methodology (equal); project administration (equal); resources (equal); writing – original draft (equal); writing – review and editing (equal). **Michael Jefford:** Conceptualization (equal); funding acquisition (equal); supervision (equal); writing – review and editing (equal). **Christopher Micheal Fisher:** Conceptualization (equal); supervision (equal); writing – review and editing (equal).

## FUNDING INFORMATION

This article is part of the INVAVICUS study, and this analysis was partially funded by the Victorian Cancer Agency, Department of Health, Victoria, Australia.

## CONFLICT OF INTEREST STATEMENT

The authors declare they have no conflicts of interest.

## Data Availability

N/A.
